# Metabolic reprogramming in polyol pathway contributes to anti-inflammatory effect of glucosamine salts on synovial fibroblasts

**DOI:** 10.1038/s44324-026-00118-0

**Published:** 2026-07-01

**Authors:** Junichi Okada, Rei Nakano, Nanako Kitanaka, Taku Kitanaka, Shinichi Namba, Masumi Nakano, Atsuto Naruke, Junichi Nunomura, Yoko Suwabe, Tadayoshi Konno, Tomoko Tohi, Satoru Kuno, Taro Kimura, Masami Uechi, Tomohiro Nakayama, Jun Yamazaki, Hiroshi Sugiya

**Affiliations:** 1 Laboratory of Veterinary Pharmacology, Nihon University College of Bioresource Sciences, Fujisawa, Kanagawa Japan; 2 Laboratory of Veterinary Radiotherapy, Nihon University College of Bioresource Sciences, Fujisawa, Kanagawa Japan; 3 Laboratory for Mucosal Immunity, RIKEN Center for Integrative Medical Sciences, Yokohama, Kanagawa Japan; 4 Japan Animal Specialty Medical Institute, Yokohama, Kanagawa Japan; 5 QIX Corporation, Machida, Tokyo Japan; 6 Kimura Animal Hospital, Shinjuku, Tokyo Japan

**Keywords:** Cell biology, Diseases, Immunology, Rheumatology

## Abstract

The upregulation of cyclooxygenase-2 (COX-2) and the subsequent production of prostaglandin E_2_ (PGE_2_) in synovial tissue are hallmarks of autoimmune arthritis, including rheumatoid arthritis (RA). While glucosamine (GlcN) derivatives modulate RA symptoms, their specific anti-inflammatory mechanisms in synovial fibroblasts (SFBs) are poorly understood. In this study, we evaluated the anti-inflammatory efficacy of various GlcN derivatives: glucosamine hydrochloride (GlcN-HCl), glucosamine sulfate (GlcN-S), glucosaminate (GlcNA), and N-acetylglucosamine (GlcNAc). GlcN-HCl and GlcN-S inhibited IL-1β-induced PGE_2_ release and the expression of COX-2 at both protein and mRNA levels, whereas GlcNA and GlcNAc exhibited no such inhibitory activity. Structure-activity relationship analysis using GlcN-HCl epimers revealed that whereas the C-4 epimer, galactosamine hydrochloride (GalN-HCl), retained potent anti-inflammatory effects, the C-2 epimer, mannosamine hydrochloride (ManN-HCl), showed significantly diminished bioactivity, highlighting the critical role of the C-2 stereochemical configuration in modulating inflammatory responses. Mechanistically, we demonstrated that GlcN-HCl-mediated COX-2 suppression occurs via epigenetic silencing rather than mRNA destabilization. GlcN-HCl treatment significantly reduced the enrichment of active chromatin marks, H3K27ac and H3K4me3, at the COX-2 promoter, whereas mRNA stability remained unaffected. Given that metabolic dysregulation is intrinsically linked to inflammatory pathogenesis, we characterized the metabolic profile of GlcN-HCl-treated SFBs. We found that GlcN-HCl triggers metabolic reprogramming of the polyol pathway by modulating the expression of *AKR1B1* and sorbitol dehydrogenase (*SORD*), resulting in elevated intracellular sorbitol levels. Pharmacological inhibition of AKR1B1 effectively abrogated the anti-inflammatory effects of GlcN-HCl, indicating that polyol pathway activation is essential for its efficacy. We confirmed the evolutionary conservation of these findings in human SFBs, demonstrating the translational relevance of the GlcN-HCl-mediated metabolic and epigenetic axis. Our findings demonstrate that GlcN-HCl induces metabolic reprogramming of the polyol pathway in synovial fibroblasts, facilitating the epigenetic silencing of inflammatory mediators. This metabolic-epigenetic axis suggests a mechanistic rationale for pharmacological metabolic intervention in RA, shifting the therapeutic paradigm toward targeted reprogramming of synovial fibroblast metabolism.

## Introduction

Rheumatoid arthritis (RA) is an autoimmune disorder characterized by chronic and progressive immune dysregulation of the synovial membrane, resulting in severe joint damage and dysfunction. The development of synovitis and joint destruction is mediated by proinflammatory cytokines, such as interleukin 1β (IL-1β) and tumor necrosis factor α (TNF-α)^1,2^. Treatment with anti-IL-1 antibodies improves RA-associated symptoms and reduces joint erosion^3,4^.

Fibroblasts are sentinel immune cells that trigger an immune response. Fibroblasts respond to several pathophysiological stimuli (*i.e*., damage-associated and pathogen-associated molecular patterns), resulting in the activation of proinflammatory signaling pathways that induce the recruitment and activation of leukocytes. In synovial tissue, synovial fibroblasts (SFBs) play a central role in synovitis by producing inflammatory mediators such as IL-1β and prostaglandins, including prostaglandin E_2_ (PGE_2_), which is a causal factor of inflammatory pain in arthritic conditions^5–7^. We previously reported that SFBs typically respond to IL-1β and produce PGE_2_ via the induction of cyclooxygenase 2 (COX-2) expression^8^.

The importance of immunometabolism in understanding the pathophysiology and development of treatments is rapidly increasing. In a previous study, changes in metabolic profiles were observed in the serum or synovial fluid of patients^9–12^. The signature of serum metabolites is correlated with an increase in the inflammatory marker, C-reactive protein^13^. Urinary signatures distinguish RA from other autoimmune diseases^14^. Differences in metabolites between RA and non-RA patients have been reported in human synovial fluid^15,16^. Increasing evidence suggests that metabolic perturbations in inflammatory states are linked to the pathophysiology of RA, and metabolites that induce anti-inflammatory metabolic reprogramming are promising tools for RA treatment^17^.

Glucosamine (GlcN) is a molecule categorized as an amino sugar formed by replacing one of the hydroxyl groups of a sugar with an amino group or a substituted amino group. In mammals, three amino sugars (GlcN, galactosamine, and neuraminic acid) are involved in various biological processes. GlcN is a substrate for glycosaminoglycan precursors (glucosamine-6-phosphate and N-acetylglucosamine [GlcNAc]). Because glycosaminoglycans are abundantly expressed on the cell surface and in the extracellular matrix of articular cartilage, GlcN is considered to promote the healing of articular cartilage via the production of glycosaminoglycans. In rodent RA models, the use of salt forms of GlcN (i.e., glucosamine hydrochloride [GlcN-HCl] and glucosamine sulfate [GlcN-S]) has been reported to attenuate RA symptoms^18–21^. In a randomized placebo-controlled study of patients with RA, GlcN treatment significantly improved pain relief^22^. Although previous studies have documented the anti-inflammatory properties of GlcN in RA, the underlying molecular mechanisms are poorly understood. The effects of GlcN on SFB inflammation remain unclear. Furthermore, despite the presence of multiple glucosamine derivatives and stereoisomers, the specific structure-activity relationship (SAR) underlying their bioactivity remains unexplored. Understanding how subtle stereochemical variations in the carbohydrate backbone dictate the anti-inflammatory responses of SFBs is essential to clarify the therapeutic potential of these compounds.

Here, we report the anti-inflammatory effects of GlcN derivatives using IL-1β-treated SFBs as a model of synovitis and the stereochemical configuration of the carbohydrate backbone, particularly at the C-2 position, plays a crucial role in its anti-inflammatory effect. Our metabolomic analysis revealed that changes in sorbitol metabolism are a characteristic of GlcN-driven metabolic reprogramming. We further validated the contribution of sorbitol to the anti-inflammatory effects of GlcN on SFBs.

## Results

### The effect of GlcN salts on IL-1β-mediated PGE_2_ release

**Fig. 1:**
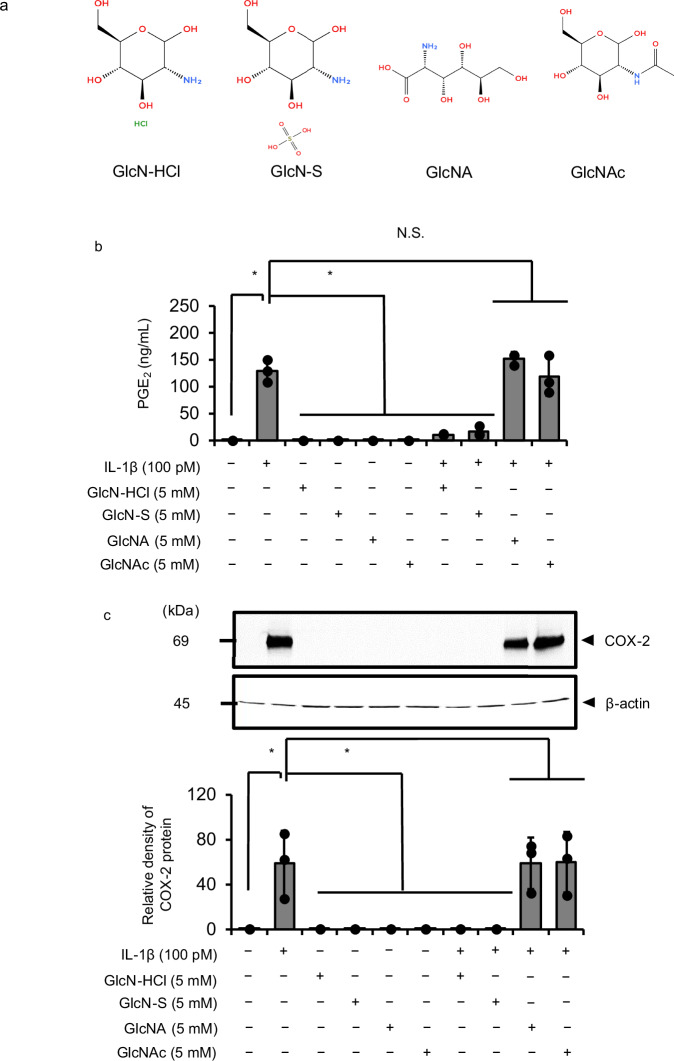
Effect of glucosamine derivatives on IL-1β-mediated PGE_2_ release. **a** Structural illustration of glucosamine and its derivatives. **b** SFBs were pretreated with 5 mM of the indicated glucosamines for 12 h and incubated with 100 pM IL-1β for 48 h. The release of PGE_2_ in the culture supernatant was measured using ELISA. GlcN-HCl and GlcN-S attenuated IL-1β-mediated PGE_2_ release. **c** When SFBs were pretreated with 5 mM of the indicated glucosamines for 12 h, the cells were incubated with 100 pM IL-1β for 48 h. GlcN-HCl and GlcN-S attenuated IL-1β-induced COX-2 protein expression. Representative blot (upper panel) and relative density of COX-2 (lower panel) compared with that of the control are shown. **a**, **c** The results are presented as mean ± SE from three independent experiments. *P < 0.05.

GlcN, which is used in pharmacological interventions, is mostly a salt derivative (i.e., GlcN-HCl and GlcN-S). In the previous studies, we reported that IL-1β (100 pM, 48 h) induced the synthesis of PGE_2_ in SFBs^8,23^. Since the production of PGE_2_ is a feature of inflammatory states in SFBs, we first examined the anti-inflammatory effects of various GlcN derivatives (*i.e*., GlcN-HCl, GlcN-S, glucosaminate [GlcNA], and GlcNAc, Fig. 1a) on IL-1β-induced PGE_2_ release. As shown in Fig. 1b, we observed a significant reduction in PGE_2_ release in the cells treated with 5 mM GlcN-HCl and 5 mM GlcN-S for 12 h; however, pretreatment with 5 mM GlcNA and 5 mM GlcNAc did not block the inflammatory effect of IL-1β. Therefore, it is likely that GlcN in salt derivatives has anti-inflammatory effects.

### The C-2 stereochemical configuration of GlcN-HCl is crucial for its anti-inflammatory activity

**Fig. 2:**
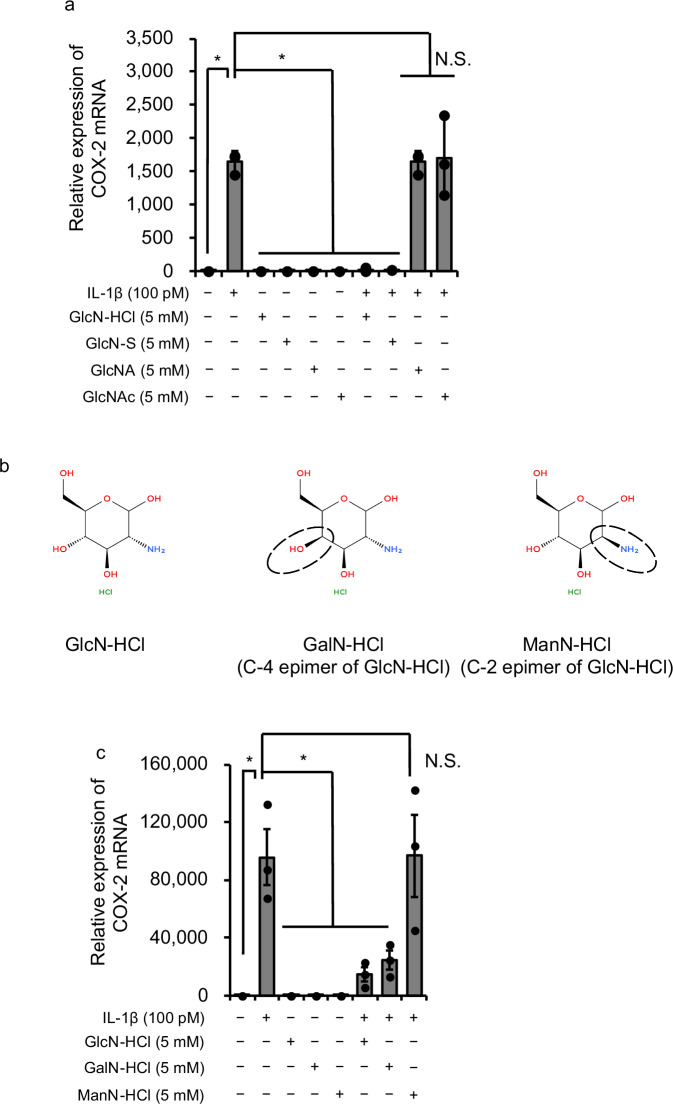
Structure-activity relationship (SAR) analysis was performed by comparing the effects of GlcN-HCl and its stereoisomers on IL-1β-induced COX-2 mRNA expression. **a** SFBs were pretreated with 5 mM of the indicated glucosamines for 12 h and incubated with 100 pM IL-1β for 48 h. GlcN-HCl and GlcN-S attenuated IL-1β-induced COX-2 mRNA expression. **b** Structural illustration of glucosamine and its stereoisomers. Dotted circles indicate the chiral centers. **c** SFBs were pretreated with 5 mM of the indicated glucosamine or its stereoisomers for 12 h and incubated with 100 pM IL-1β for 48 h. GlcN-HCl and GalN-HCl attenuated IL-1β-induced COX-2 mRNA expression, whereas ManN-HCl had no effect. **a**, **c** Results are presented as mean ± SE from three independent experiments. *P < 0.05.

The rate-limiting enzyme in the synthesis of PGE_2_ is COX (i.e., the constitutive form, COX-1, and the inducible form, COX-2). In synovial fibroblasts, the synthesis of PGE_2_ is regulated by the induction of COX-2 expression^8,23^. We investigated whether GlcN derivatives attenuated the induction of COX-2 mRNA and protein expression. IL-1β (100 pM) for 48 h induced COX-2 mRNA expression in SFBs, as reported previously^8,23^. We evaluated the effects of GlcN-HCl, GlcN-S, GlcNA, and GlcNAc on IL-1β-induced COX-2 protein expression. As shown in Fig. 1c, treatment with 5 mM GlcN-HCl or GlcN-S significantly attenuated COX-2 protein levels, whereas GlcNA and GlcNAc at the same concentration had no effect. This inhibitory pattern was consistent at the mRNA level; both GlcN-HCl and GlcN-S suppressed IL-1β-induced COX-2 mRNA expression, whereas GlcNA and GlcNAc failed to inhibit that (Fig. 2a). Dose-response analysis further confirmed that GlcN-HCl (Fig. S1a) and GlcN-S (Fig. S1b) exerted their inhibitory effects in a dose-dependent manner, whereas GlcNA (Fig. S1c) and GlcNAc (Fig. S1d) showed no significant dose-dependent inhibition. We also confirmed that the mRNA expression of COX-1 remained stable in IL-1β-treated cells with or without 5 mM GlcN derivatives (Fig. S2a). Cell viability remained unaffected across all GlcN derivatives at concentrations of up to 10 mM (Fig. S3a–d), ensuring that the observed anti-inflammatory effects were not caused by cytotoxicity. These observations support the notion that GlcN-HCl or GlcN-S negatively regulates PGE_2_ production by inhibiting COX-2 expression at the mRNA level. To delineate the structural requirements for the bioactivity of GlcN-HCl, we performed a SAR analysis by comparing GlcN-HCl with stereoisomers, specifically the C-4 epimer galactosamine hydrochloride (GalN-HCl) and the C-2 epimer mannosamine hydrochloride (ManN-HCl) (Fig. 2b). Despite their close structural similarities, these epimers displayed markedly different regulatory profiles. While GalN-HCl retained potent anti-inflammatory effects comparable to those of GlcN-HCl, epimeric substitution at the C-2 position (ManN-HCl) caused a near-complete loss of inhibitory activity toward COX-2 mRNA expression (Fig. 2c). Dose-response analysis further confirmed that both GlcN-HCl and GalN-HCl exerted inhibitory effects in a dose-dependent manner (Fig. S4a). In contrast, ManN-HCl exhibited no significant dose-dependent inhibition, even at higher concentrations (Figure. S4a). Cell viability remained stable in the presence of GlcN-HCl (Fig. S3a), GalN-HCl (Fig. S4b, left panel), and ManN-HCl (Fig. S4b, right panel) at concentrations up to 10 mM. These findings demonstrate that the stereochemical configuration of the carbohydrate backbone, particularly at the C-2 position, is a critical determinant of the modulation of inflammatory responses in SFBs.

### GlcN-HCl induced epigenetic alteration of active histone mark at the COX-2 promoter

**Fig. 3:**
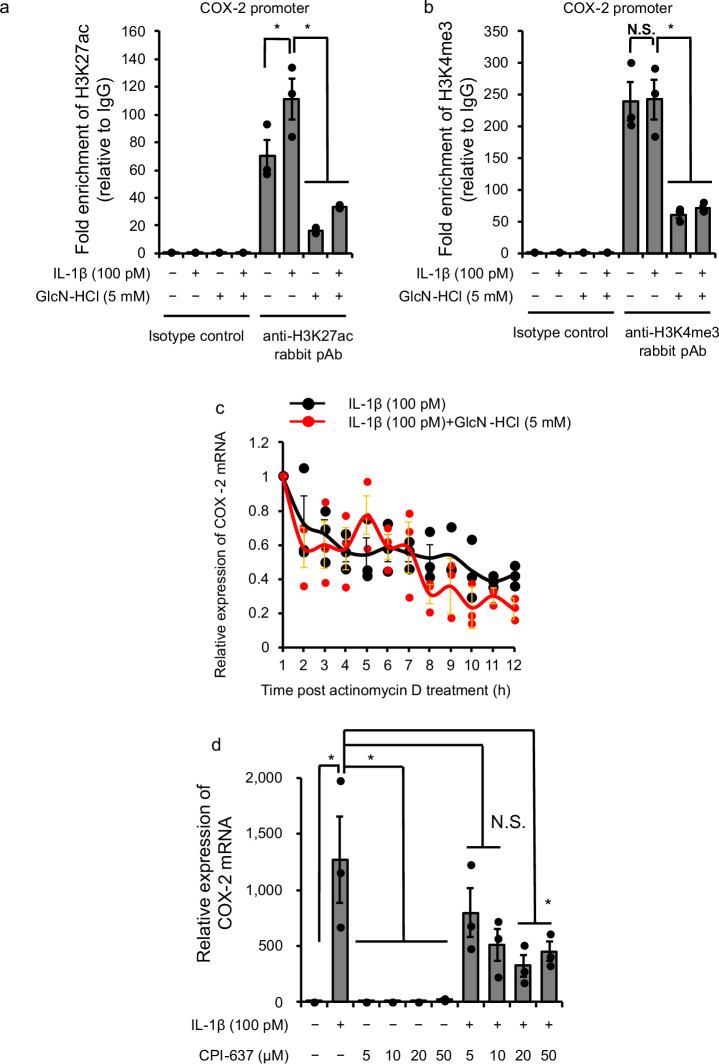
GlcN-HCl inhibits the enrichment of active histone marks at the COX-2 promoter and a histone acetyltransferase inhibitor attenuates IL-1β-induced COX-2 mRNA expression. **a**, **b** SFBs were pretreated with 5 mM GlcN-HCl for 12 h and incubated with 100 pM IL-1β for 48 h. The first four bars represent non-specific binding (Isotype control), while the subsequent four bars show enrichment for the specific histone marks H3K27ac (**a**) and H3K4me3 (**b**). Enrichment of H3K27ac (**a**) and H3K4me3 (**b**) at the COX-2 promoter was detected by chromatin immunocleavage coupled with qPCR. GlcN-HCl significantly reduced the enrichment of active histone marks, specifically H3K27ac (**a**) and H3K4me3 (**b**), at the COX-2 promoter region. (**c**) The stability of IL-1β-induced COX-2 mRNA was detected using an actinomycin D (ActD)-based pulse-chase assay followed by RT-qPCR. The cells were incubated with 100 pM IL-1β for 6 h with or without 12 h of 5 mM GlcN-HCl pretreatment. Subsequently, the cells were treated with 10 μM ActD and RNA was harvested 1-12 h after ActD treatment. GlcN-HCl did not affect the stability of IL-1β-induced COX-2 mRNA. **d** IL-1β-induced COX-2 mRNA expression was inhibited by the histone acetyltransferase inhibitor CPI-637. After pretreatment of CPI-637 (0-50 μM) for 1 h, the cells were stimulated with 100 pM IL-1β for 48 h. CPI-637 dose-dependently attenuated IL-1β-induced COX-2 mRNA expression. **a**–**d** Results are presented as mean ± SE from three independent experiments. *P < 0.05.

We previously reported that activation of the mitogen-activated protein kinase (MAPK) pathways (i.e., ERK1/2 and p38 activation) stimulates IL-1β-mediated COX-2 mRNA transcription and PGE_2_ release in primary-cultured SFBs^8^. The activation of NF-κB pathway (i.e., p65 activation) has also been reported as a transcriptional factor for IL-1β-mediated COX-2 mRNA expression^23–25^. Thus, we first examined the effect of GlcN-HCl (5 mM) on IL-1β-induced ERK1/2 phosphorylation (Fig. S5a and b), p38 (Fig. S5c and d), and p65 (Fig. S5 e, f), suggesting that its inhibitory effect involves regulatory mechanisms independent of the MAPK and NF-κB pathways. To delineate alternative mechanisms, we analyzed the impact of GlcN-HCl on epigenetic modifications and mRNA stability. Our results demonstrated that GlcN-HCl significantly reduced the enrichment of active histone marks, specifically H3K27ac (Fig. 3a) and H3K4me3 (Fig. 3b) in the COX-2 promoter. In contrast, actinomycin D-based pulsed-chase assays revealed that COX-2 mRNA stability was unaffected by GlcN-HCl treatment (Fig. 3c). To clarify the functional link between H3K27ac to COX-2 transcriptional regulation, we applied the histone acetyltransferase inhibitor CPI-637 (0 to 50 µM, 1 h). As shown in Fig. 3d, CPI-637 dose-dependently attenuated the IL-1β-induced COX-2 mRNA expression. Collectively, these findings indicate that GlcN-HCl-mediated COX-2 suppression occurs primarily through epigenetic silencing at the transcriptional level rather than through mRNA destabilization.

### The metabolic reprogramming by GlcN-HCl targets sorbitol metabolism

**Fig. 4:**
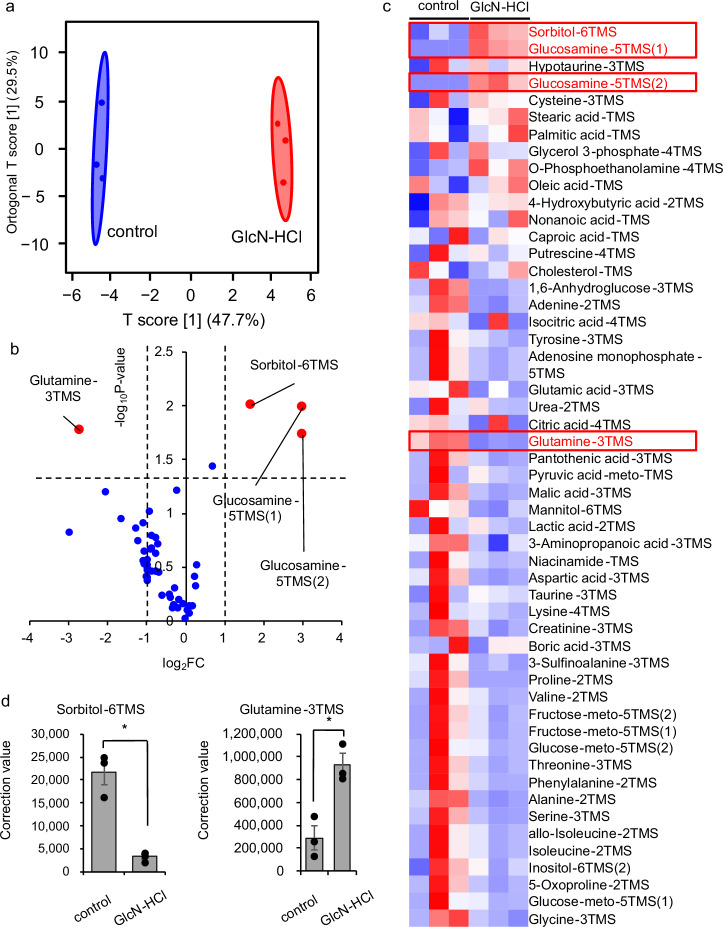
GlcN-HCl induces metabolic reprogramming of SFBs into an anti-inflammatory phenotype. When SFBs were treated with 5 mM GlcN-HCl for 12 h, metabolome analysis was performed using GCMS. **a** Orthogonal partial least squares-discriminant analysis (OPLS-DA) showed global changes in the metabolic features of SFBs treated with GlcN-HCl. **b** Changes in metabolites in GlcN-HCl-treated cells compared to the control. The horizontal dotted line indicates the threshold (-log_10_ *P* value = 1.3) for statistical significance, equivalent to *P* = 0.05, and the vertical dotted line indicates the threshold for the fold change (FC) of metabolites ( | log_2_ FC | = 1), equivalent to an |FC | = 2 (**b**). A significant increase in glucosamine and sorbitol levels and a significant decrease in glutamine levels were observed. **c** Heatmaps of differential metabolites between GlcN-HCl and control groups. **d** Changes in sorbitol (left panel) and glutamine (right panel) levels in GlcN-HCl-treated cells compared to the control. **d** The results are presented as mean ± SE from three independent experiments. *P < 0.05.

Immunometabolic shifts play a pivotal role in RA pathogenesis. To elucidate the mechanisms underlying the anti-inflammatory action of GlcN-HCl, we characterized the metabolic landscape of GlcN-HCl-treated SFBs. The cells were then washed with ice-cold PBS and treated with 60% perchloric acid solution. The cell lysate was centrifuged (4 °C, 15000 *× g*, 10 min), and the pH was adjusted to 7.4 using KOH. After centrifugation (4 °C, 15000 *× g*, 10 min), the supernatant (100 μL) was collected and subjected to intracellular metabolome analysis. The orthogonal partial least squares-discriminant analysis (OPLS-DA) showed that the metabolic profiles of GlcN-HCl–treated SFBs were clearly distinct from those of controls, supporting the notion that extracellular GlcN-HCl drives metabolic reprogramming toward an anti-inflammatory phenotype (Fig. 4a). The cross-validated cumulative modeled variations R2X, R2Y, and Q2 coefficients of the predictive loading (p1) and orthogonal (o1, o2) components (Fig. S6). We detected an increase in intracellular GlcN levels in GlcN-HCl-treated cells, suggesting that increased intracellular GlcN-HCl contributes to metabolic reprogramming (Fig. 4b, c). Furthermore, GlcN-HCl treatment induced a significant increase in sorbitol levels and a decrease in L-glutamine levels (Fig. 4b–d). Over-representation analysis using the Relational database of Metabolomic Pathways (RaMP-DB) showed significant enrichment of pathways related to sorbitol (e.g., fructose biosynthesis and the polyol pathway, Fig. S7 upper panel) or L-glutamine (e.g., Defective GFPT1 causes CMSTA1 and Sterol regulatory element-binding proteins (SREBP) signaling, Fig. S7 lower panel). These observations suggest that changes in metabolite levels play a crucial role in the anti-inflammatory effects of GlcN-HCl.

### Sorbitol attenuates IL-1β-mediated COX-2 expression

**Fig. 5:**
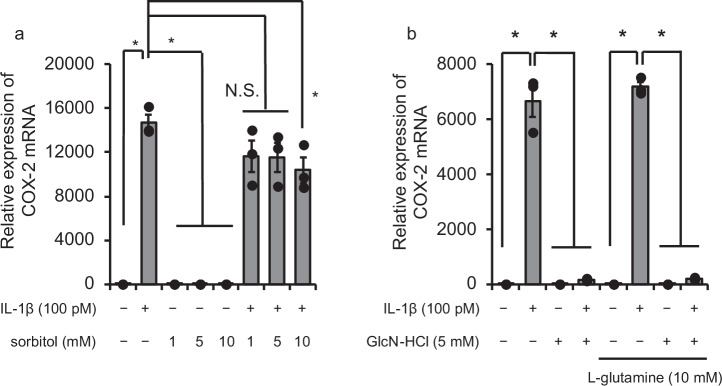
Sorbitol attenuated IL-1β-induced COX-2 mRNA expression. **a** SFBs were pretreated with 0–10 mM sorbitol for 12 h and incubated with 100 pM IL-1β for 48 h. Sorbitol attenuated IL-1β-induced COX-2 mRNA expression in a dose-dependent manner. **b** SFBs were pretreated with 10 mM glutamine for 12 h with or without 5 mM GlcN-HCl, and the cells were incubated with 100 pM IL-1β for 48 h. Glutamine failed to recover the GlcN-HCl-dependent attenuation of IL-1β-induced COX-2 mRNA expression. **a**, **b** The results are presented as mean ± SE from three independent experiments. *P < 0.05.

We then investigated whether GlcN-induced metabolic reprogramming contributed to the reduction in IL-1β-induced COX-2 expression. As an increase in sorbitol levels was detected in GlcN-HCl-treated cells, we investigated the effect of sorbitol on IL-1β-induced COX-2 expression. Sorbitol enhanced ADP- and collagen-induced aggregation in platelets at a concentration of 10 mM^26^. During in vitro maturation of oocytes 20 mM sorbitol stimulates blastocyst development and improves embryo quality, whereas 100 mM sorbitol induces osmotic stress^27^. These observations suggest that approximately 10 mM sorbitol can be used in treatments without causing osmotic stress. Consistent with previous reports, we confirmed that sorbitol at concentrations up to 10 mM had no adverse effects on SFB viability (Fig. S8). Based on the cell viability assay, we pretreated SFBs with 0–10 mM sorbitol for 12 h and subsequently incubated the cells with IL-1β for 6 h. We observed that IL-1β-induced COX-2 expression was significantly attenuated in cells pretreated with 10 mM sorbitol (Fig. 5a). We investigated the involvement of L-glutamine in the anti-inflammatory effects of GlcN-HCl. Given that L-glutamine levels decreased in GlcN-HCl-treated cells, we investigated whether L-glutamine counteracted the inhibitory effect of GlcN-HCl on IL-1β-induced COX-2 expression^28,29^. As shown in Fig. 5b, 10 mM L-glutamine did not inhibit the anti-inflammatory effect of GlcN-HCl. Taken together, these observations suggest that sorbitol plays a dominant role in the anti-inflammatory action of GlcN-HCl on SFBs.

### The effect of GlcN-HCl on the expression of sorbitol metabolic enzymes

**Fig. 6:**
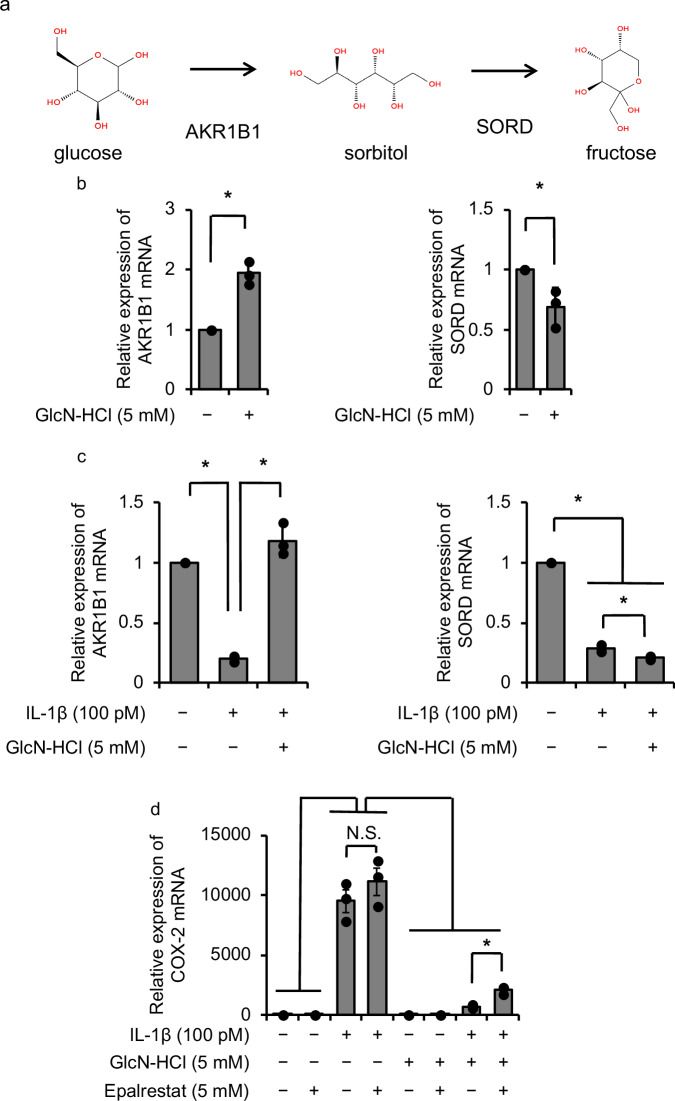
GlcN-HCl induces changes in the mRNA expression of the polyol pathway enzymes AKR1B1 and SORD. **a** Schematic illustration of the polyol pathway. **b** SFBs were treated with 5 mM GlcN-HCl for 12 h, and the mRNA expression levels of AKR1B1 and SORD were detected using RT-qPCR. AKR1B1 mRNA expression significantly increased in GlcN-HCl-treated SFBs, whereas SORD mRNA expression decreased. **c** SFBs were pretreated with 5 mM GlcN-HCl for 12 h and incubated with 100 pM IL-1β for 48 h. IL-1β significantly reduced AKR1B1 mRNA expression, which was recovered by GlcN-HCl treatment. Both IL-1β and GlcN-HCl significantly reduced SORD mRNA expression. **d** SFBs were pretreated with or without 50 μM Epalrestat (an AKR1B1 inhibitor) for 12 h, followed by treatment with 5 mM GlcN-HCl for 12 h. Subsequently, the cells were stimulated with 100 pM IL-1β for 48 h. In the presence of Epalrestat, the suppressive effect of GlcN-HCl on IL-1β-induced COX-2 expression was partially attenuated. **b**–**d** Results are presented as mean ± SE from three independent experiments. *P < 0.05.

Sorbitol is primarily metabolized via the polyol pathway. In the polyol pathway, glucose is converted to sorbitol by aldose reductase AKR1B1, followed by the conversion of sorbitol to fructose by SORD (Fig. 6a). We examined the effects of GlcN-HCl on AKR1B1 and SORD mRNA expressions. As shown in Fig. 6b, GlcN-HCl increased the mRNA expression of AKR1B1 and reduced that of SORD, supporting GlcN-HCl-mediated metabolic reprogramming in sorbitol metabolism. In the presence of IL-1β, we observed a reduction in AKR1B1 mRNA expression, which was recovered by treatment with GlcN-HCl (Fig. 6c). IL-1β also mediated a decrease in the mRNA expression of SORD, which was further reduced by GlcN-HCl treatment (Fig. 6c). To validate the functional necessity of this pathway, we used an inhibitor of AKR1B1 Epalrestat to SFBs. In the presence of the AKR1B1 inhibitor, the effect of GlcN-HCl was partially attenuated (Fig. 6d). This partial reversal was consistent with our observations following direct sorbitol supplementation. Taken together with our metabolomic analysis, GlcN-HCl induces metabolic reprogramming in the polyol pathway via the induction of AKR1B1 and reduction of SORD expression, which increases sorbitol levels. To determine whether these metabolic shifts were secondary to generalized metabolic stress or nutrient-sensing pathways, we examined the effects of the AMPK activator AICAR. In contrast to GlcN-HCl treatment, AICAR treatment did not alter AKR1B1 or SORD mRNA expression (Fig. S9), suggesting that GlcN-HCl regulates the polyol pathway through a specific structural mechanism rather than via the canonical AMPK signaling pathway.

### GlcN-HCl-mediated inhibition of IL-1β-induced COX-2 mRNA expression via the modulation of the polyol pathway is translationally conserved in human synovial fibroblasts

**Fig. 7:**
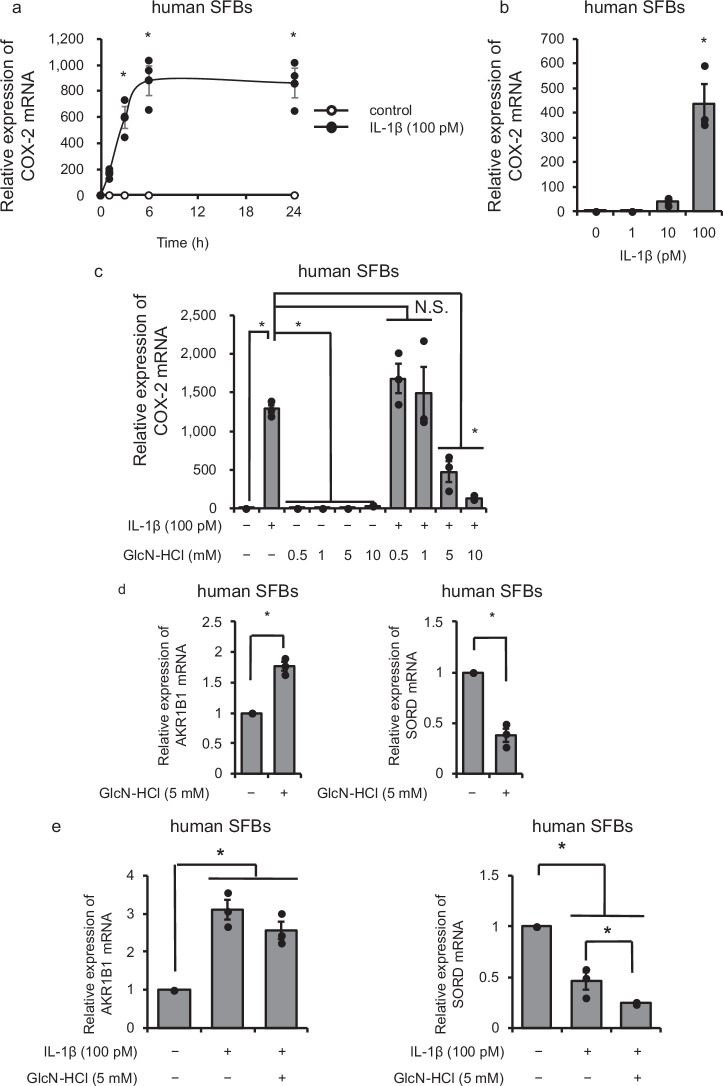
Consistency of the effect of GlcN-HCl on IL-1β-induced COX-2 mRNA expression in human synovial fibroblasts. **a** Human SFBs were treated with 100 pM IL-1β for the time indicated, and the mRNA expression of COX-2 was detected using RT-qPCR. IL-1β induced COX-2 mRNA expression in a time-dependent manner. **b** Human SFBs were treated with 0–100 pM IL-1β for 24 h. IL-1β induced COX-2 mRNA expression in a dose-dependent manner. **c** Human SFBs were pretreated with 0–10 mM GlcN-HCl for 12 h and incubated with 100 pM IL-1β for 24 h. GlcN-HCl attenuated IL-1β-induced COX-2 mRNA expression. **d** Human SFBs were treated with 5 mM GlcN-HCl for 12 h, and the mRNA expression of AKR1B1 and SORD was detected using RT-qPCR. AKR1B1 mRNA expression significantly increased in GlcN-HCl-treated SFBs, whereas SORD mRNA expression decreased. **e** Human SFBs were pretreated with 5 mM GlcN-HCl for 12 h and incubated with 100 pM IL-1β for 24 h. AKR1B1 mRNA expression significantly increased in both IL-1β-treated and IL-1β/GlcN-HCl-treated human SFBs, whereas SORD mRNA expression decreased in IL-1β/GlcN-HCl-treated human SFBs compared to that in IL-1β-treated. **a**–**e** The results are presented as mean ± SE from three independent experiments. *P < 0.05.

To validate the interspecies conservation of this metabolic mechanism, we extended our investigation to include human SFBs. To clarify the basis for the experimental conditions, we examined the effect of IL-1β on COX-2 mRNA expression in human SFBs. When human SFBs were treated with 100 pM IL-1β, the mRNA expression of COX-2 increased in a time-dependent manner (Fig. 7a). IL-1β induced COX-2 mRNA expression in a dose-dependent manner (Fig. 7b). Based on the experiments for time- and dose-dependent effects of IL-1β on human SFBs, we investigated the anti-inflammatory effect of GlcN-HCl on 100 pM IL-1β-induced COX-2 mRNA expression. As shown in Fig. 7c, GlcN-HCl inhibited IL-1β-induced COX-2 mRNA expression in a dose-dependent manner. Mechanistically, GlcN-HCl upregulated AKR1B1 (Fig. 7d, left panel) and downregulated SORD mRNA expression (Fig. 7d, right panel) in human SFBs, mirroring the shifts observed in a feline model. Interestingly, IL-1β suppressed AKR1B1 mRNA expression in feline cells, while it increased AKR1B1 mRNA levels in human SFBs (Fig. 7e). However, GlcN-HCl treatment consistently maintained high AKR1B1 expression levels compared to control in both species (Fig. 7e, left panel). Conversely, IL-1β reduced SORD expression, and this reduction was further reduced by GlcN-HCl (Fig. 7e, right panel), thus promoting a metabolic environment conducive to sorbitol accumulation. To confirm the clinical relevance, we analyzed publicly available data from RA patient synovial tissue (GSE12021, control *n* = 4 vs RA *n* = 11). Consistent with our in vitro observations, signal intensity of PTGS2 mRNA and AKR1B1 mRNA significantly increased in RA synovial tissue compared to controls, while that of SORD mRNA showed no significant change (Fig. S10). These observations support the translational relevance of GlcN-HCl-mediated anti-inflammatory effects and the underlying polyol pathway regulation.

## Discussion

In this study, we demonstrated that GlcN salt derivatives (GlcN-HCl and GlcN-S) attenuated the effect of IL-1β on PGE_2_ release. Mechanistically, our results suggest that the decrease in PGE_2_ release can be attributed to a reduction in PGE_2_ production via the attenuation of COX-2 expression at the transcriptional level. Furthermore, GlcN-HCl-driven metabolic reprogramming increased intracellular sorbitol levels, contributing to its anti-inflammatory effects. Therefore, GlcN in the salt form appears to be the most suitable for alleviating the effects of IL-1β on the synovium.

Although clinical trials have reported that GlcN significantly improves pain relief in patients with RA, the underlying molecular mechanisms remain unclear^22^. Our study bridges the gap between clinical observations and cellular metabolic reprogramming. By comparing various GlcN derivatives, including GlcN-HCl, GlcN-S, GlcNA, and GlcNAc, we found that the basic salt forms (GlcN-HCl and GlcN-S) had anti-inflammatory efficacy. SAR analysis showed that the requirement for a specific stereochemical configuration at the C-2 position. Despite the minimal structural divergence between GlcN-HCl and its C-2 epimer ManN-HCl, the latter failed to suppress COX-2 expression. This structural stringency provides a plausible basis for the anti-inflammatory action of GlcN-HCl, whereas the modification of the carbohydrate backbone in GlcNA and GlcNAc is associated with a loss of inhibitory efficacy. The inability of ManN-HCl to mimic the effects of GlcN-HCl further supports our hypothesis that GlcN-HCl acts as a specific metabolic ligand, rather than as a mere nutrient source. Our epigenetic analysis provides insights into the molecular mechanisms underlying this anti-inflammatory response of GlcN-HCl. GlcN-HCl suppressed *COX-2* mRNA expression, which was associated with alterations in the epigenetic marks at the promoter region. Specifically, GlcN-HCl inhibited the enrichment of active histone marks H3K27ac and H3K4me3 at the COX-2 promoter. These alterations in the epigenetic landscape suggest that GlcN-HCl promotes a less active chromatin environment, potentially attenuating the induction of pro-inflammatory gene expression.

Although inflammatory signaling pathways are important components of RA pathophysiology, inducing remission using conventional therapies targeting specific signaling pathways remains difficult. Understanding metabolic reprogramming in SFBs will provide valuable insights into the pathophysiology and development of anti-inflammatory therapies for RA^30^. mTOR inhibitors (i.e., rapamycin, sirolimus, everolimus, and temsirolimus) and the AMPK activator/mitochondrial Complex I inhibitor metformin have been reported to be promising metabolism-targeting agents for RA.^17^. Teriflunomide, the active metabolite of leflunomide, attenuates mitochondrial function and de novo pyrimidine synthesis by inhibiting dihydroorotate dehydrogenase activity^17^. Methotrexate, a folic acid analog that inhibits purine and pyrimidine synthesis, has also been reported as a promising agent for RA treatment via the induction of adenosine accumulation^24^. Based on previous reports, we focused on the metabolism of GlcN-HCl-treated SFBs. Our metabolomic analysis demonstrated that GlcN-HCl treatment shifted the metabolic profile of SFBs, resulting in a clear statistical separation from the control group. Our metabolomic analysis revealed a significant increase in intracellular sorbitol levels after GlcN-HCl treatment. Sorbitol has been reported to act as a cytotoxic osmolyte in glucose-sensitive tissues, such as the nerves and retina. However, our results suggest that sorbitol acts as a protective anti-inflammatory metabolite in synovial tissue. This discrepancy suggests tissue-specific resilience in SFBs. The biological impact of a metabolite is not inherent to the molecule itself but is dictated by the metabolic landscape of the target tissues. Although excess sorbitol is a stressor, it can function as a chemical chaperone at a regulated concentration. The accumulation of sorbitol could facilitate protein stability via preferential exclusion, a process characterized by an increase in the chemical potential of the protein-surface hydration layer^31^. This biophysical role prevents protein misfolding and aggregation, which are often exacerbated by inflammation. In contrast to neurons, which are sensitive to glucose metabolism and oxidative stress, SFBs appear to possess markedly higher thresholds for osmotic and metabolic fluctuations. Although absolute intracellular sorbitol concentrations were not quantified in this study, the selection of 10 mM for our mechanistic experiments was based on the fold-increase observed in our metabolomic profiling and established physiological ranges for organic osmolytes, which had no effect on the cell viability. In the unique environment of the joint, characterized by osmotic and mechanical fluctuations, the accumulated sorbitol could potentially act as a chemical chaperone, contributing to cellular homeostasis. Further studies are required to quantify the intracellular sorbitol levels in SFBs of RA patients.

Although IL-1β-induced H3K27ac and H3K4me3 are reduced by GlcN-HCl, the precise molecular mechanism has been unclear. Previous reports have shown that the histone acetyltransferase p300/CBP is sensitive to the intracellular metabolic state, including local acetyl-CoA availability and the cellular redox balance^32,33^. The consumption of NADPH and potential modulation of the NAD^+^/NADH ratio may affect the enzymatic activity of p300 or regulate its interaction with cofactors^25^. We hypothesized that GlcN-HCl-induced accumulation of sorbitol, or its immediate metabolic impact (such as shifts in the NAD^+^/NADH or NADP^+^/NADPH ratios), may interfere with IL-1β-induced histone epigenetic modification in SFBs by suppressing the recruitment of histone deacetylases, such as NAD^+^-dependent sirtuin, further favoring a repressive deacylated chromatin state^34^. By bypassing canonical signaling cascades (MAPK/NF-κB) and directly remodeling the chromatin landscape, GlcN-HCl provides a sustained epigenetic brake on inflammatory gene transcription. Further studies are needed to clarify the precise molecular mechanisms of GlcN-HCl on epigenetic regulation.

The anti-inflammatory properties of GlcN-HCl have been attributed to the suppression of specific signaling pathways. GlcN-HCl was reported to inhibit p38 MAPK phosphorylation in the CSABI-479 cell line^35^. We have also previously demonstrated that ERK1/2 and p38 are essential mediators of IL-1β-induced COX-2 mRNA expression in primary SFBs^8^. However, in the current study, GlcN-HCl did not affect ERK1/2 or p38 phosphorylation levels. These observations suggest that the anti-inflammatory effect of GlcN-HCl in SFBs is unlikely to be predominantly caused by attenuated MAPK activation. Our metabolomic analysis revealed a significant increase in intracellular GlcN levels, indicating an intracellular metabolic mechanism. This shift was accompanied by distinct changes in the expression of polyol pathway genes (*i.e*., AKR1B1 and SORD). The regulation of these enzymes is complex, and previous studies have identified binding regions for TonEBP/NFAT5, AP-1, and androgen-responsive elements within the regulatory regions of the *AKR1B1* gene in both mice and humans^36^. Androgens have been reported to regulate *SORD* expression in human prostate cancer cell lines^37^. Although our data suggests that intracellular GlcN-HCl acts as a modulator of polyol pathway enzymes, specific transcription factors linking intracellular GlcN to the anti-inflammatory response are under investigation in our laboratory.

In this study, we extended our investigation to validate the interspecies conservation of the anti-inflammatory properties of GlcN-HCl. In our study, GlcN-HCl upregulated AKR1B1 and downregulated SORD mRNA expression in human SFBs, which is consistent with the results observed in our feline SFBs model. The reduction of sorbitol catabolic enzyme SORD, combined with the maintenance of AKR1B1, likely facilitates the metabolic flux toward sorbitol. GlcN-HCl treatment consistently attenuated IL-1β-induced COX-2 mRNA expression in both human and feline SFBs. This interspecies consistency underscores that the GlcN-HCl-mediated metabolic-epigenetic axis is a fundamental biological mechanism, rather than a species-specific phenomenon. Taken together with our re-analysis of the public dataset from synovial tissues of patients with RA, these observations support our mechanistic observations and highlight the potential of GlcN-HCl as a translational therapeutic approach. By targeting a conserved metabolic-epigenetic axis, this strategy contributes to the development of metabolism-based disease-modifying therapies for RA and related inflammatory joint diseases in both human and veterinary medicine.

In conclusion, our results suggest that the C-2 stereochemical configuration of GlcN-HCl is linked to the reduction of IL-1β–induced COX-2 expression and PGE_2_ production, alongside alterations in sorbitol metabolism. These findings indicate that metabolic shifts may play a role in inflammatory responses and could represent potential therapeutic strategies for inflammatory joint disease.

## Methods

### Materials

TRIzol reagent was purchased from Life Technologies (Carlsbad, CA, USA). GlcN-HCl, GlcN-S, GlcNA, GlcNAc, and Epalrestat were purchased from MedChemExpress (Monmouth Junction, NJ, USA). GalN-HCl and ManN-HCl were purchased from Tokyo Chemical Industry Co. Ltd. (Tokyo, Japan). CPI-637 was obtained from Sellck Chemicals (Houston, TX, USA). Cell Counting Kit-8 was purchased from Dojindo (Kumamoto, Japan). NucleoSpin Gel and PCR Cleanup, Thermal Cycler Dice Real-Time System II, TP900 Dice RealTime v4.02B, SYBR Premix Ex Taq II, PrimeScript RT Master Mix, and CELLBANKER 1 Plus Medium were purchased from TaKaRa Bio Inc. (Shiga, Japan). Rabbit monoclonal antibodies against human cyclooxygenase-1 (COX-1; EPR5867) and rabbit polyclonal antibodies against COX-2 and H3K27ac were purchased from Abcam (Cambridge, UK). Mouse monoclonal anti-β-actin antibody (AC74) and actinomycin D were purchased from Sigma-Aldrich Inc. (St Louis, MO). ChIC/Cut&Run assay kit, anti-H3K4me3 (C42D8), anti-rat total-ERK1/2 (t-ERK1/2, 137F5), anti-human phospho-ERK1/2 (p-ERK1/2, D13.14.4E), anti-human total-p38 (t-p38, D13E1), anti-human phospho-p38 (p-p38, 3D7), anti-phosphorylated p65 (p-p65, 93H1), and anti-total p65 (t-p65, D14E12) rabbit monoclonal or polyclonal antibodies were obtained from Cell Signaling Technology Japan, K.K. (Tokyo, Japan). Amersham ImageQuant 8000 was purchased from Global Life Sciences Technologies Japan K.K. (Tokyo, Japan). Horseradish peroxidase (HRP)-conjugated anti-mouse and anti-rabbit IgG antibodies and an ECL western blotting analysis system were obtained from GE Healthcare (Piscataway, NJ, USA). Mini-PROTEAN TGX gels, polyvinylidene difluoride (PVDF) membranes, and iCyclers were purchased from Bio-Rad (Hercules, CA, USA). Block Ace and Complete mini EDTA-free protease inhibitor mixtures were purchased from Roche (Basel, Switzerland). Dulbecco’s modified Eagle medium (DMEM) supplemented with 1 g/L glucose was obtained from Wako Pure Chemical Industries Ltd. (Osaka, Japan). An enzyme-linked immunosorbent assay (ELISA) kit for PGE_2_ was obtained from Cayman Chemical Co. (Ann Arbor, MI, USA). The freezing vessel (BICELL) was purchased from Nihon Freezer Co. Ltd. (Tokyo, Japan). Human synovial fibroblasts were purchased from Articular Engineering, LLC. (Northbrook, IL, USA).

### Cell culture for feline SFBs

This study was approved by the Institutional Animal Care and Use Committee of Kimura Animal Hospital (approval numbers: KAH2014-001, KAH2015-001, and KAH2015-002). All experiments were performed in accordance with the guidelines and regulations of Kimura Animal Hospital. Informed consent was obtained from all the owners. Feline synovial membranes were obtained from the healthy knee joints of domestic short-haired cats during reductive surgery for femoral fractures (*n* = 3, 4–7 years old, spayed females). Animals were treated with atropine sulfate (0.05 mg/kg, subcutaneously; Mitsubishi Tanabe Pharma Co., Osaka, Japan). Propofol (4.0 mg/kg, intravenous injection; Intervet K. K., Osaka, Japan) was administered to induce anesthesia. 2.0% isoflurane (Intervet K.K.) and 100% oxygen were administered through an endotracheal tube to maintain anesthesia during the procedure. To control pain and infection, intravenous administration of remifentanil hydrochloride (3–5 µg/kg/min; Janssen Pharmaceutical K.K, Tokyo, Japan) and cefazolin (22 mg/kg; Nichi-Iko Pharmaceutical Co., Ltd, Toyama, Japan) was performed before awakening. Feline SFBs were isolated from synovial membranes by explant culture using a previously described method with a few modifications^23,38,39^.

### GlcN treatment for feline SFBs

Feline SFBs were pretreated with 5 mM or the indicated concentration of GlcN derivatives for 12 h, and the cells were incubated with 100 pM IL-1β for 48 h. For drug treatment, SFBs were pretreated with or without the AMPK activator AICAR (100 μM, 1 h) or the histone acetyltransferase inhibitor CPI-637 (5 to 50 µM, 1 h), and then stimulated with 100 pM IL-1β for 48 h. In SFBs pretreated with or without the AKR1B1 inhibitor Epalrestat (50 μM, 12 h), the cells were further treated with 5 mM GlcN-HCl for 12 h, and then stimulated with 100 pM IL-1β for 48 h. Total RNA samples were collected using the TRIZOL reagent. Total protein samples were collected using lysis buffer (20 mM HEPES at pH 7.4, complete mini EDTA-free protease inhibitor cocktail, 1 mM PMSF, and 10 mM sodium fluoride).

### Cell culture for human SFBs

Human SFBs from the synovial membrane of healthy donors were maintained in static culture in an incubator at 5% CO_2_ and 37 °C using DMEM with 10% fetal bovine serum. The medium was replaced twice a week. Human SFBs were harvested using 0.25% trypsin-EDTA once they reached 90-95% confluence. After suspension, the cells were collected using CELLBANKER 1 Plus Medium at a density of 2 × 10^6^ cells/500 µL. The cell suspension was then placed in sterilized serum tubes. The tubes were then placed in a BICELL vessel and cryopreserved at −80 °C. The serum tubes were removed from the BICELL vessel and immersed in a 37 °C water bath. Before the experiments, the thawed cell suspension was transferred to a centrifuge tube containing DMEM with 10% fetal bovine serum and centrifuged at 500 × *g* for 2 min. The supernatant was removed, and the pellet was suspended in DMEM with 10% fetal bovine serum and transferred to a 10-cm culture dish. Static cultures were maintained under the same conditions as before the cryopreservation. The cells were harvested using 0.25% trypsin-EDTA once they reached approximately 90% confluence. The collected cells were seeded at a density of 1 × 10^6^ cells per 10-cm culture dish. The human SFBs used for all experiments were between passages six to eight.

### GlcN treatment for human SFBs

Human SFBs were pretreated with the indicated concentrations of GlcN derivatives for 12 h, and the cells were incubated with 100 pM IL-1β for 24 h. Total RNA samples were collected using the TRIZOL reagent.

### Cell viability assay

The cells were seeded at a density of 5000 cells/well in a 96-well culture plate. After GlcN treatment, cell viability was measured using Cell Counting Kit-8, according to the manufacturer’s instructions.

### Real-time reverse transcription-polymerase chain reaction (RT-qPCR)

TRIzol reagent was used to extract the total RNA from SFBs. First-strand cDNA was synthesized from total RNA (500 ng) using PrimeScript RT Master Mix^40–43^. RT-qPCR was performed with 2 µL of the first-strand cDNA, SYBR Premix Ex Taq II, and primers specific for AKR1B1, COX-2, COX-1, SORD, and β-actin (a housekeeping gene used as an internal control) (see Table 1 and 2) in a total reaction volume of 25 μL^8^. No-template controls and no-reverse transcription controls were performed using 2 µL of RNase- and DNA-free water and each RNA sample, respectively. The following PCR protocol was run with the Thermal Cycler Dice Real-Time System II: denaturation (95 °C, 30 s), 40 cycles of denaturation (95 °C, 5 s), and annealing/extension (60 °C, 30 s). The second derivative maximum method and the comparative cycle threshold (ΔΔCt) method were performed using real-time RT-PCR analysis software. Amplification of β-actin or TATA-box binding protein (TBP) from the same amount of cDNA was used as an endogenous control for feline or human SFBs, respectively. The cDNA amplified from the SFBs at time 0 was used as a calibration standard.

**Table 1:** Primer sequences for quantitative reverse transcription-polymerase chain reaction (qRT-PCR)

Gene Name	Gene bank ID	F or R	Primer sequences (5’ to 3’)
*feline COX-1*	XM_003995827	F	ggacaacctggaacgtcagt
		R	aaacacctcctgacccacag
*feline COX-2*	EF036473.1	F	aacaggagcatccagaatgg
		R	gcagctctgggtcaaacttc
*feline AKR1B1*	XM_003983075.5	F	ccgagaacttccaggtctttga
		R	accgacggcttcaaaactca
*feline SORD*	XM_003987197.6	F	gatattgcaacacgtggccg
		R	ggcttccagagctttctcca
*feline β-actin*	AB051104.1	F	ctcttccagccttccttcct
		R	gacagcaccgtgttagcgta
human *COX-2*	NM_000963.4	F	cggtgaaactctggctagacag
		R	gcaaaccgtagatgctcaggga
human *AKR1B1*	NM_001628.4	F	tactcagctacaacaggaactg
		R	aggcaagaaacacaggtatagg
human *SORD*	NM_003104.6	F	actccagagccaaaagagc
		R	catcctcagcaagacctcat
human *TBP*	NM_003194.5	F	tgctgcggtaatcatgaggata
		R	tgaagtccaagaacttagctggaa

**Table 2:** Primer sequences for chromatin immunocleavage coupled with qPCR (Chic-qPCR)

Target	F or R	Sequence (5’- > 3’)	Position	gene ID
COX-2 promoter	F	TCATGTCGTCACGTGGGCTT	21762854 - 21762873	NC_058384.1
	R	ACACTCGGGAACTTCGCACA	21762967 - 21762948	

### Chromatin immunocleavage coupled with qPCR (Chic-qPCR)

Chic-qPCR was performed using a ChIC/Cut&Run assay kit, according to the manufacturer’s instructions. Briefly, cells (5 × 10^5^/assay) were collected using 0.25% trypsin-EDTA. The digitonin-treated cells (100 μL/assay) were mixed with activated concanavalin A-conjugated beads (10 μL/assay) and incubated at 4 °C for 18 h with antibodies (Rabbit (DA1E) Monoclonal Antibody IgG Isotype Control [0.5 μg/assay], anti-H3K4me3 (C42D8) rabbit mAb [0.25 μg/assay], anti-H3K27ac rabbit pAb [1 μg/assay]). After incubation, the samples were incubated with pAG-MNase at 4 °C for 1 h. After washing three times with digitonin buffer, the target chromatin was digested by adding CaCl_2_. Chromatin digestion was stopped using 1 × STOP buffer. The digested target chromatin was purified using NucleoSpin Gel and PCR Cleanup kits. The enrichment of H3K27ac and H3K4me3 relative to IgG was detected by qPCR using 1 ng of purified chromatin DNA per reaction.

### Actinomycin D (ActD)-based pulse-chase assay followed by RT-qPCR

Feline SFBs were seeded at a density of 3 × 10^5^ cells/well in 6-well culture plate. To assess mRNA stability, the cells were pretreated with 5 mM GlcN-HCl for 12 h, followed by stimulation with 100 pM IL-1β for 6 h. Subsequently, 10 μM ActD was added to the medium to inhibit de novo transcription of the gene. Total RNA was harvested hourly from 0 to 12 h after ActD treatment for subsequent RT-qPCR analysis.

### Western blotting

Western blotting was performed as previously described^37–49^. To collect protein samples, lysis buffer (20 mM HEPES at pH 7.4, complete mini EDTA-free protease inhibitor cocktail, 1 mM PMSF, and 10 mM sodium fluoride) was used. The Bradford method was used to adjust the protein concentration^50^. The extracted proteins were boiled at 95 °C for 5 min in 3 × SDS/DTT buffer. Samples were electrophoretically separated on 7.5% or 12% polyacrylamide gels and transferred to PVDF membranes. The membrane was incubated with a blocking agent for 50 min at 25 °C. Primary antibodies [β-actin (1:10,000, a housekeeping gene used as internal control), p-p38 (1:1000), t-p38 (1:1000), p-p65 (1:1000), and t-p65 (1:1000),COX-2 (1:1000), COX-1 (1:100), p-ERK1/2 (1:1000), t-ERK1/2 (1:1000)] were applied to treat the membrane for 120 min 25 °C. The membranes were washed and treated with secondary antibodies (HRP-conjugated anti-mouse or anti-rabbit IgG antibodies [1:10,000]) for 90 min at 25 °C. Chemiluminescent signals were detected using the ECL western blotting analysis system and Amersham ImageQuant 8000.

### ELISA for PGE_2_

Feline SFBs (3.0 × 10^5^ cells/well) were seeded in six-well culture plates^8^. After starvation for 24 h, the fibroblasts were treated with IL-1β for 48 h, and the culture medium was collected. PGE_2_ concentration in the culture medium was determined using an ELISA kit according to the manufacturer’s instructions.

### Gas chromatography-mass spectrometry (GCMS) analysis for metabolome

Feline SFBs were treated with 60% perchloric acid, and 100 μL of the supernatant samples were collected. Methoxyamine hydrochloride, pyridine, and N-methyl-N-trimethylsilyltrifluoroacetamide were used for oxime conversion and trimethylsilylation, respectively. GCMS was performed using a GCMS-QP2010 Ultra instrument (Shimadzu). The derivatized metabolites were separated using a DB-5 column (30 m × 0.25 mm id; film thickness, 1.0 mm; Agilent Technologies). The flow rate of the He carrier gas was 39 cm/s. The inlet temperature was 280 °C, and the column temperature was first held at 100 °C for 4 min, then raised at 4 °C/min to 320 °C, and held for 8 min. Approximately 0.5 μL of the sample was injected into the GCMS in splitless mode. The mass spectra were obtained under the following conditions: electron ionization (ionization voltage, 70 eV); ion source temperature, 200 °C; interface temperature, 280 °C; full scan mode in the range of m/z 45–600; and scan rate, 2000 µ/s. Deconvolution and component detection were performed using the Analyzer Pro (Thermo Fisher Scientific). Metabolite estimation was performed using FragmentAlign (Shimadzu). Metabolomic data were processed and analyzed using the web-based platform MetaboAnalyst 6.0 (ref. ^51^, https://www.metaboanalyst.ca). Over-representation analysis was performed using the Reactome pathway database (RaMP-DB^52^) based on significantly altered metabolites ( | log_2_ fold change (FC)| ≥ 1 and –log_10_ *P* > 1.3).

### Re-analysis of the public dataset

The publicly available data from synovial tissue of patients with RA (GSE12021, control *n* = 4 vs RA *n* = 11) were reanalyzed using GEO2R^53^.

### Statistical analysis

All statistical analyses were performed using the EZR software^54^. Data are shown as mean ± standard error and were analyzed using one-way analysis of variance (ANOVA) with the Holm-Bonferroni multiple comparison test as a post hoc test. Statistical significance was set at *P* < 0.05.

## Supplementary information

Supplementary Information

## Data Availability

All data supporting the findings of this study are available in this manuscript.
