# Supplementary material for: Metabolic reprogramming in polyol pathway contributes to anti-inflammatory effect of glucosamine salts on synovial fibroblasts
Source: NPJ Metab Health Dis. 2026 Jul 1;4:24. doi: 10.1038/s44324-026-00118-0 (PMC13324759; doi:10.1038/s44324-026-00118-0)
Supplement: Supplementary file 1 — Supplementary Information [file 44324_2026_118_MOESM1_ESM.pdf]

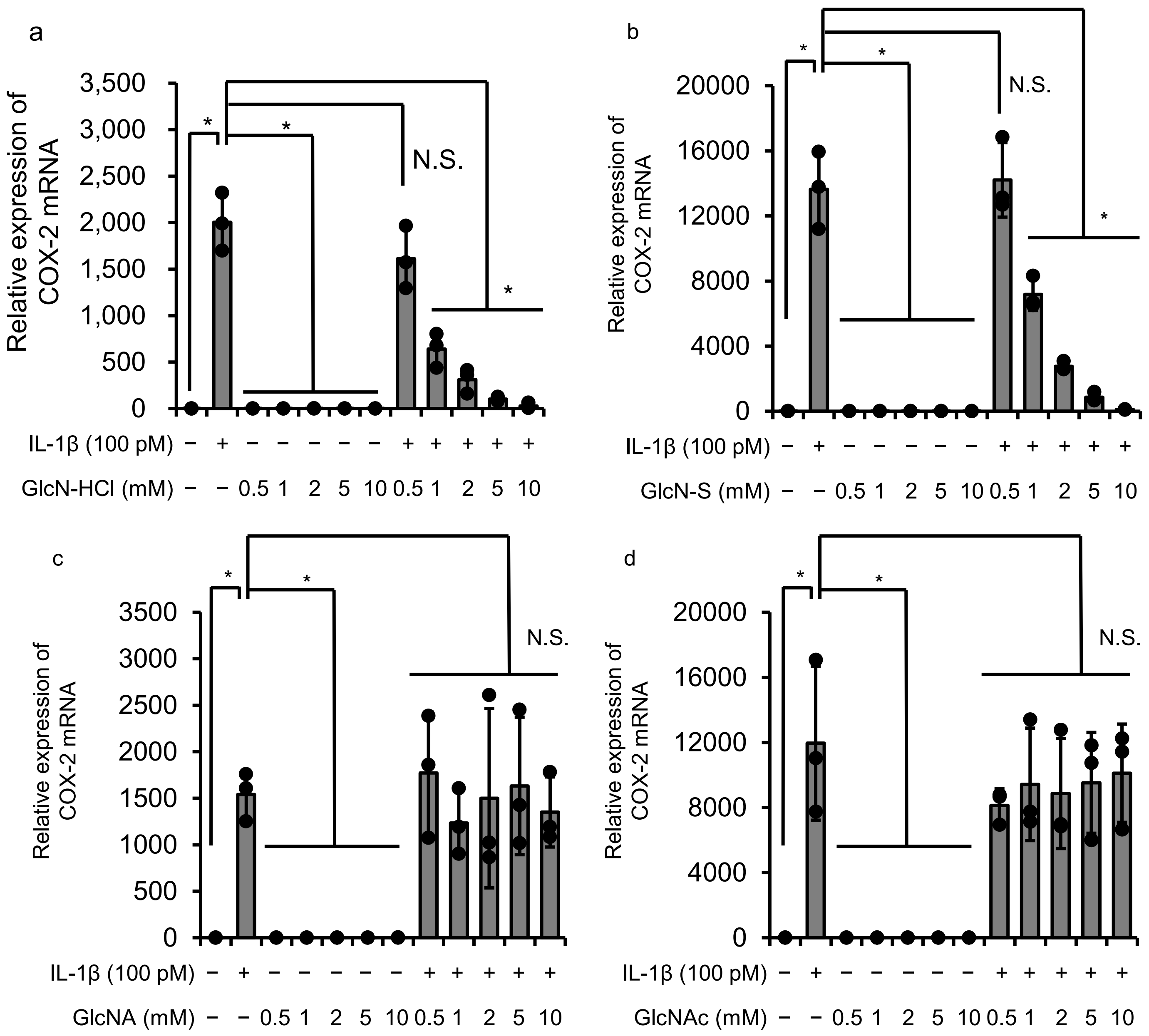

Fig. S1. Dose-dependent effect of GlcN-HCl, GlcN-S, GlcNA and GlcNAc on COX-2 mRNA expression in SFBs. Results are presented as mean ± SE from 3 independent experiments. *P<0.05.

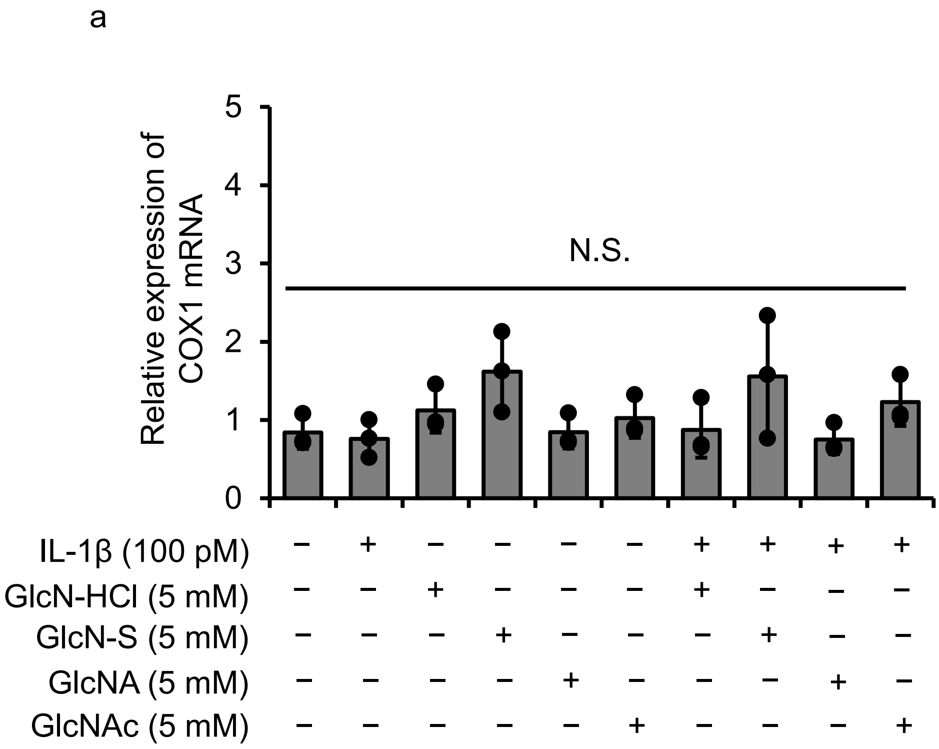

Fig. S2. No effect of GlcN-HCl on COX-1 mRNA expression in SFBs. Results are presented as mean ± SE from 3 independent experiments. *P<0.05.

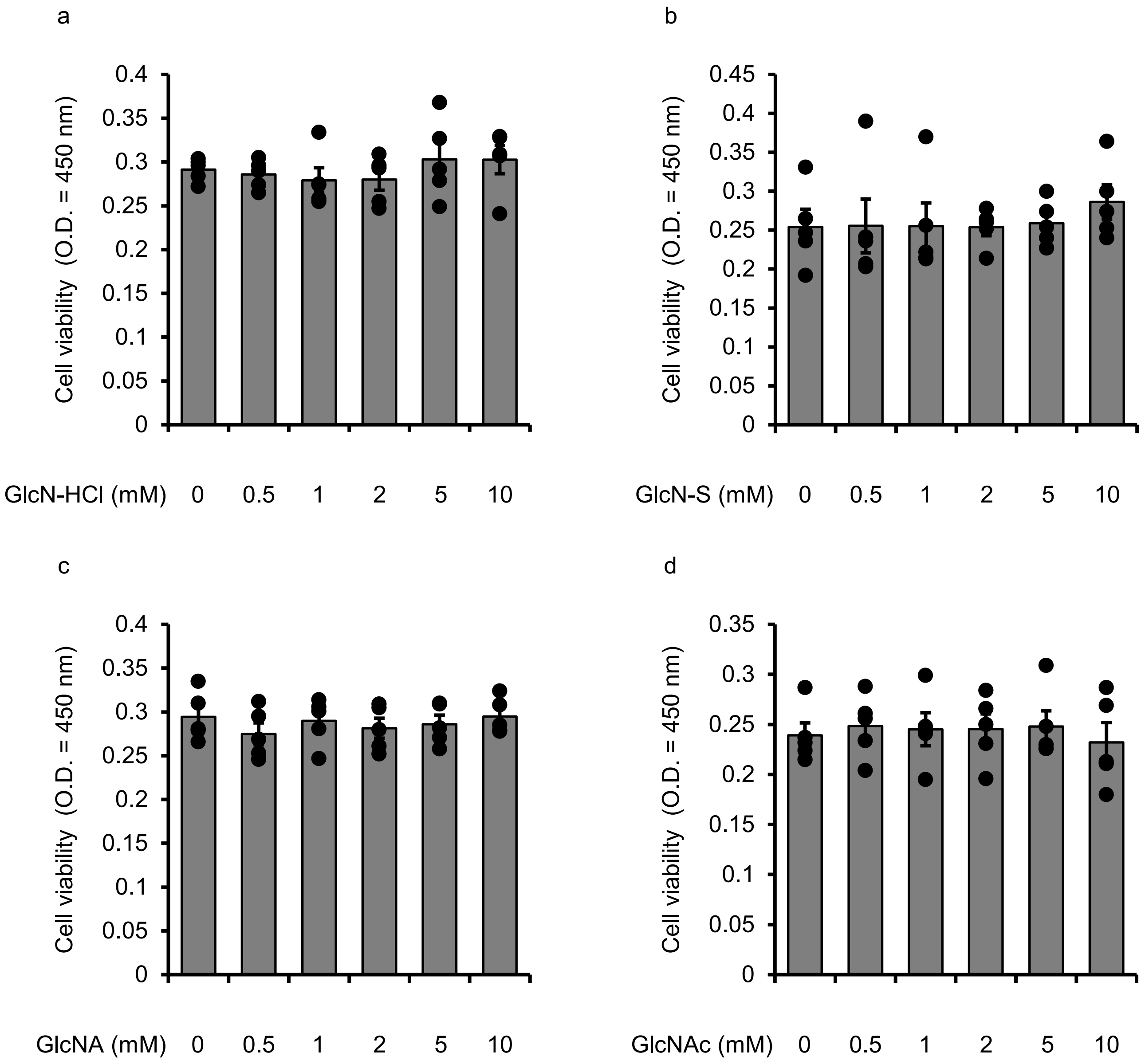

Fig. S3. The viability of SFBs remained stable in the presence of GlcN-HCl, GlcN-S, GlcNA and GlcNAc. Results are presented as mean ± SE from 5 independent experiments.

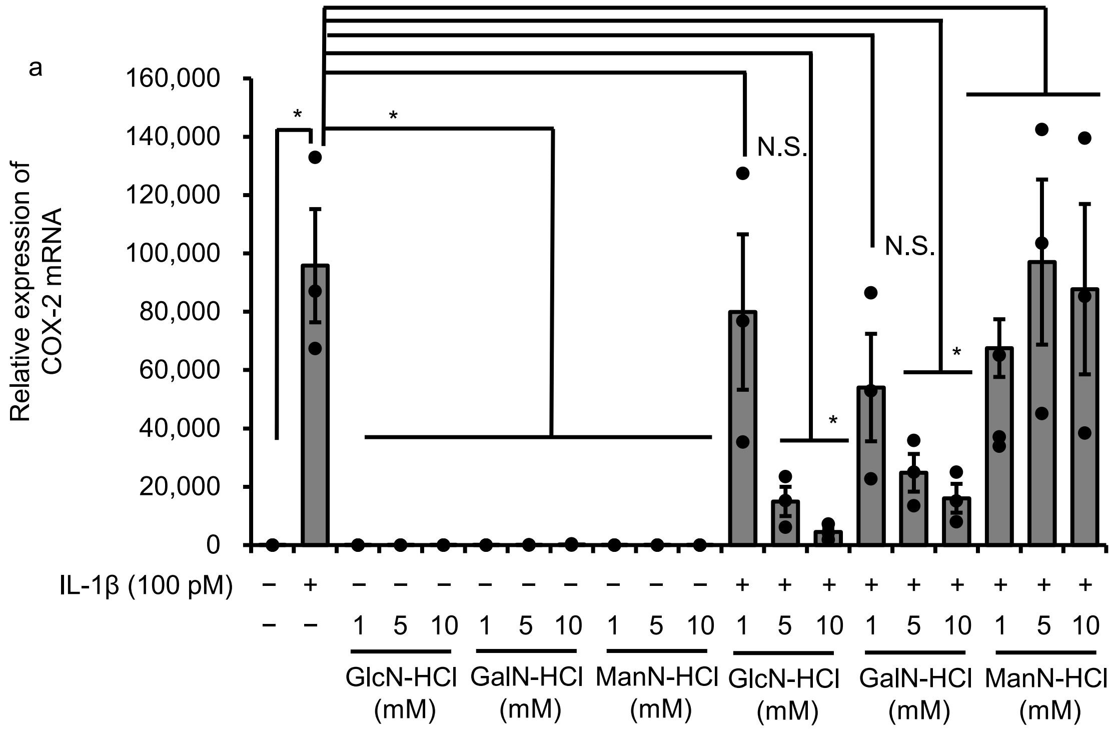

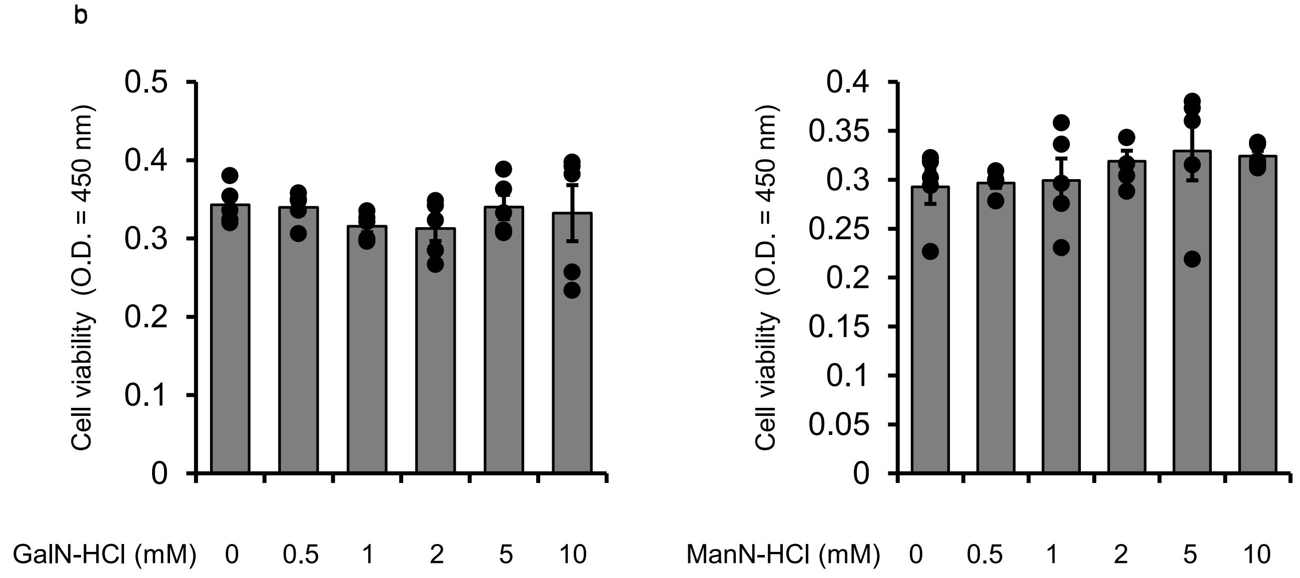

Fig. S4. (a) Dose-dependent effect of GlcN-HCl, GalN-HCl and ManN-HCl on COX-2 mRNA expression in SFBs (b) Dose-dependent effect of GalN-HCl (left panel) and ManN-HCl (right panel) on cellular viability in SFBs. Results are presented as mean ± SE from 3 independent experiments. *$P<0.05$.

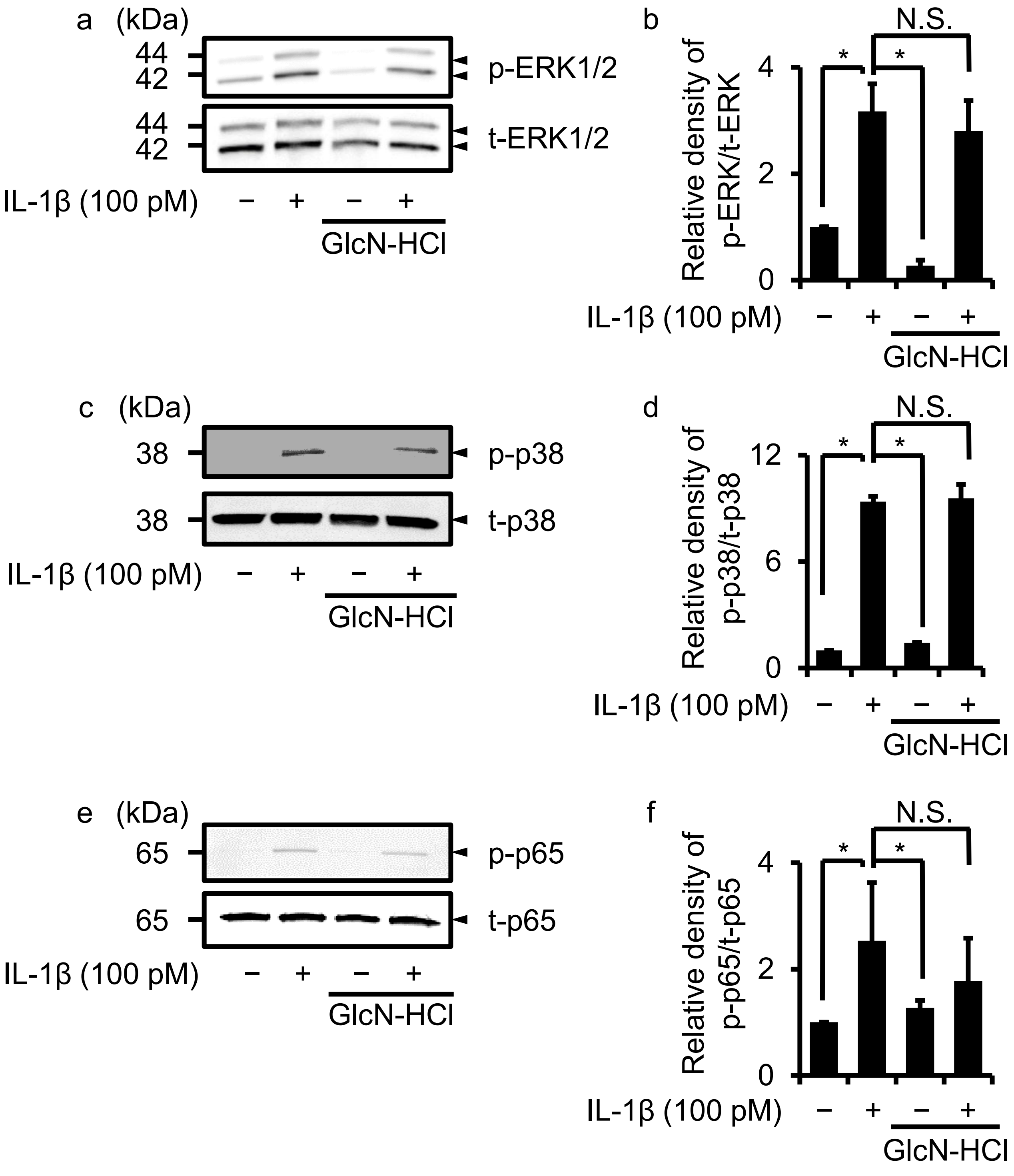

Fig. S5. No effect of GlcN-HCl on the phosphorylation of ERK1/2, p38 and p65 in SFBs. Representative blot (a, c and e) and relative density (b, d, and f) compared with control are shown. Results are presented as mean ± SE from 3 independent experiments. **P*<0.05.

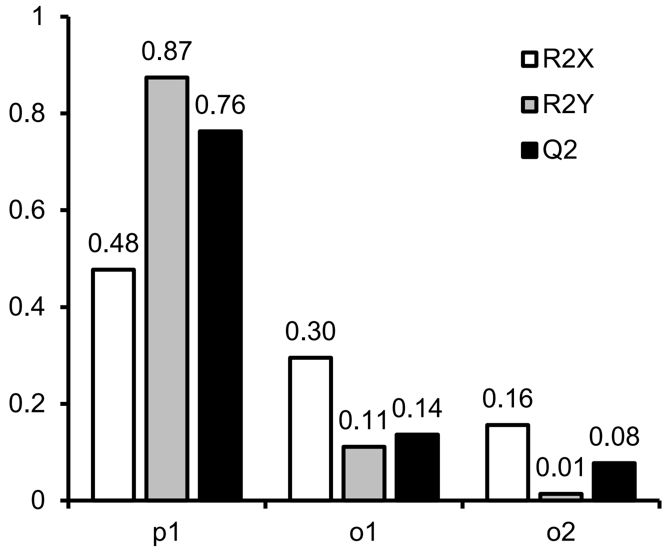

Fig. S6. Parameters of orthogonal partial least squares-discriminant analysis (OPLS-DA) comparing GlcN-HCl-treated and control groups. The predictive component (p1) accounted for the majority of class discrimination, while orthogonal components (o1 and o2) captured non-discriminatory variance. The model exhibited strong explanatory and predictive performance (R2X = 0.48, R2Y = 0.87, Q2 = 0.76).

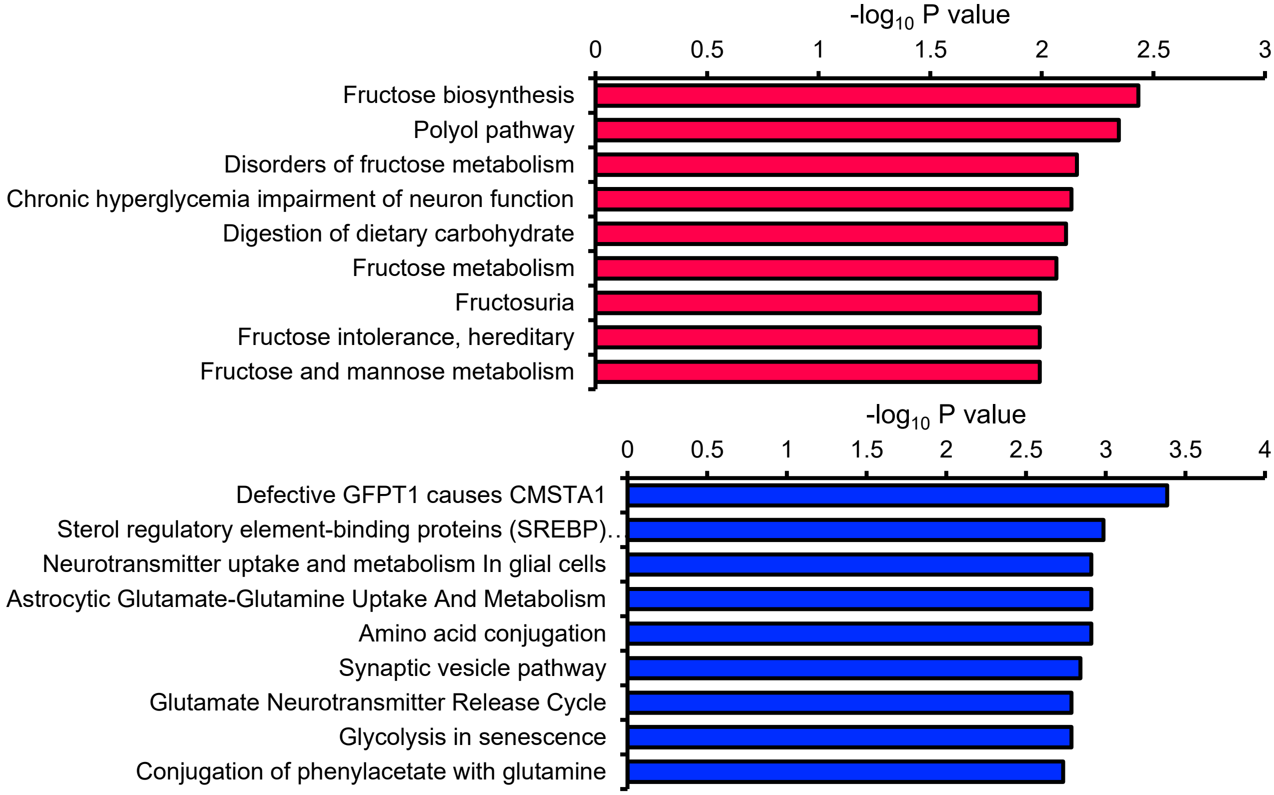

Fig. S7. Over-representation analysis based on the Relational database of Metabolomic Pathways (RaMP-DB). Red bars indicate pathways enriched with significantly increased metabolites in the GlcN-HCl group ($\log_2$ fold change ≥ 1 and $-\log_{10} P > 1.3$), whereas blue bars indicate pathways enriched with significantly decreased metabolites in the GlcN-HCl group ($\log_2$ fold change ≤ -1 and $-\log_{10} P > 1.3$).

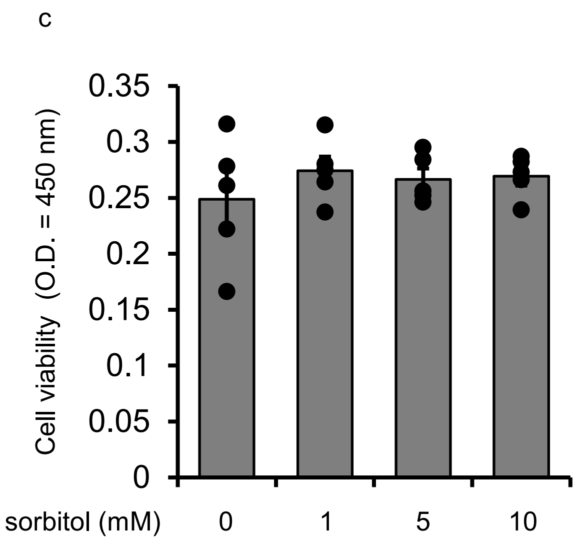

Fig. S8. The viability of SFBs remained stable in the presence of sorbitol. Results are presented as mean ± SE from 5 independent experiments.

a

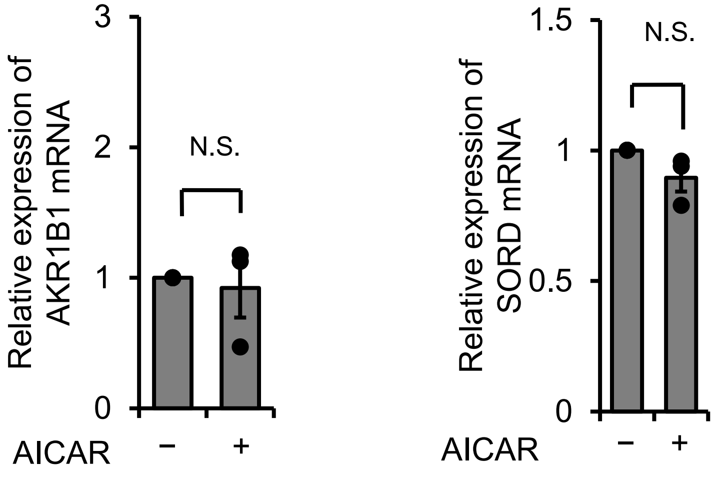

Fig. S9. No effect of AICAR on the mRNA expression of *AKR1B1* and *SORD* in SFBs. Results are presented as mean ± SE from 3 independent experiments.

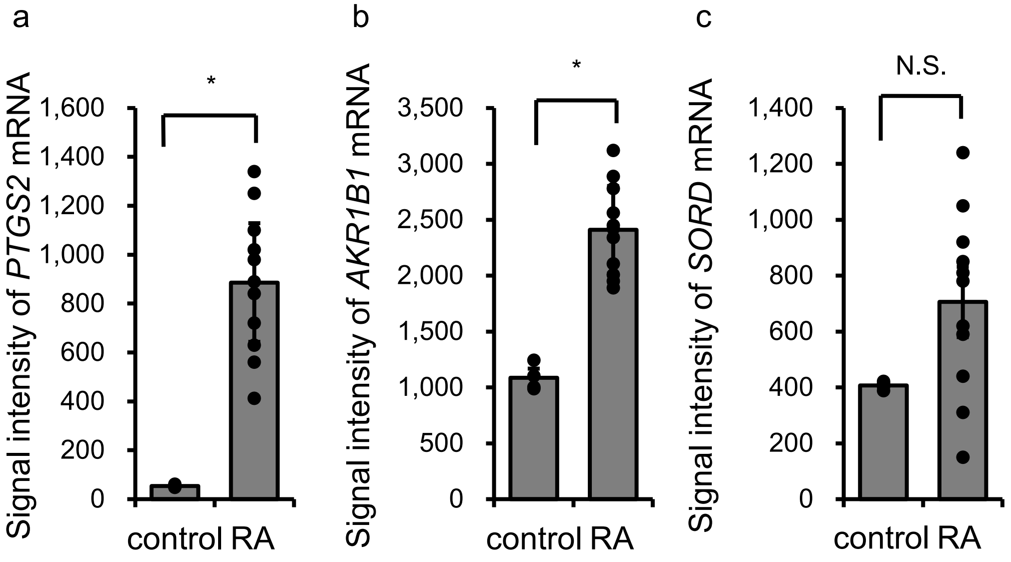

Fig. S10. Validation of the polyol pathway in human RA samples. Re-analysis of the public dataset GSE12021 (Healthy control, n = 4; RA patients, n = 11). The mRNA levels of PTGS2 (a) and AKR1B1 (b) are significantly elevated in the synovial tissue of RA patients compared to healthy controls, while SORD mRNA level (c) remained stable. Data are presented as mean ± SEM. Statistical significance was determined by unpaired two-tailed Student's t-test; $*P < 0.05$.

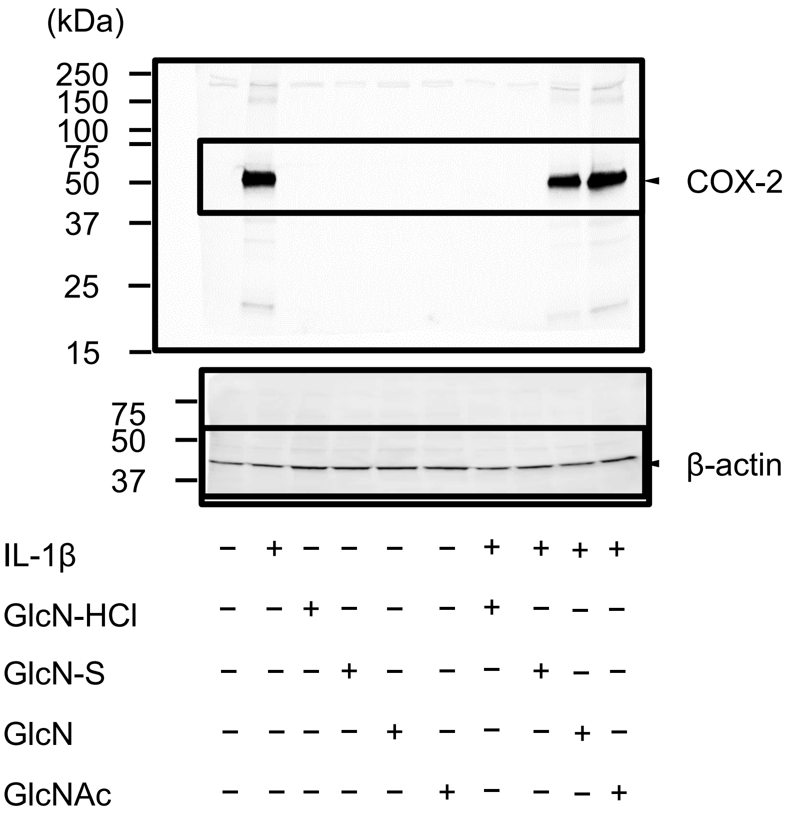

Fig. S10. Uncropped images for the blots shown in Fig. 2a.
